# Changes in Selected Parameters of Swimming Technique in the Back Crawl and the Front Crawl in Young Novice Swimmers

**DOI:** 10.2478/hukin-2013-0037

**Published:** 2013-07-05

**Authors:** Damian Jerszyński, Katarzyna Antosiak-Cyrak, Małgorzata Habiera, Krystian Wochna, Elżbieta Rostkowska

**Affiliations:** 1 Poznan University of Medical Sciences, Poznań, Poland.; 2 Academy of Physical Education, Poznań, Poland.

**Keywords:** stroke cycle, angles, shoulders roll, distance per stroke, swimming

## Abstract

The study aimed to examine changes in selected angular characteristics and duration of the stroke cycle in the back crawl and the front crawl in children learning to swim. Nine boys and two girls, aged 8–13 years, performed seven consecutive swimming tests. The children’s movement technique was recorded with the use of three video cameras. The studied parameters included the angle of incidence between the trunk long axis and the waterline, elbow angle, shoulders roll, stroke cycle duration and stroke length. The results illustrate the development of swimming technique in youth swimmers. The results of the present study indicate the variability and phasing of learning of swimming technique by children.

## Introduction

The movements of particular body parts during swimming have been artificially developed in the form of four swimming styles. Since the modern swimming strokes are not natural movements, learning them is a long process and requires permanent error correction. Each novice swimmer should, however, be allowed some degree of individualism in learning movement technique, which would account for individual somatic and physiological differences.

Studies involving video recording of swimming techniques, turns and starts were carried out by researchers from the United States, Australia, Great Britain, Korea, Germany, Norway, Japan and Poland ( Kjendlie et al., 2004 , 2008 , 2004 ). Their methodology was based on a comparison of anthropometric indices, buoyancy, body roll and swimming technique between children (12 years of age) and adults (21 years of age). The authors noted that adults mastered movement technique better, while body roll was greater and more visible in children. Thompson (2007) studied biomechanical aspects of the elbow angle values in swimmers. Payton et al. (1999) , as well as Seifert et al. (2007) , concentrated on the coordination between arm and leg movements and breathing as well as body roll. Also Haljand (1997) and Yoshizawa (1982) examined biomechanical parameters in elite swimmers, using synchronized surface and underwater video cameras. The results of these studies allowed instructors and swimmers to introduce changes in swimming technique, which ultimately contributed to the improvement of swimming performance ( Haljand, 1997 ; Yoshizawa, 1982 ).

Improvement of swimming technique has been a major factor in swimming training for many years. Literature abounds in works documenting the dynamic development of swimming technique research. A study by Seyfried (2007) from the National Sport Institute in Paris revealed that an increment in swimming velocity is directly related to an increase in stroke length and a decrease in stroke rate. The study results were used for development of NATAVIT and AQUACYCLE training enhancing software ( Syfried, 2007 ).

At present, the most common method of analysis of a swimmer’s movements is video recording of particular movement stages using underwater cameras. Havriluk (2009) studied swimmers’ movements using footage from an underwater camera synchronized with respective hand force diagrams. He distinguished three factors limiting the swimmer’s efficiency: differences between the right arm and left arm, loss of force and redundant movements. These factors can be observed in all swimmers, including elite ones ( Havriluk, 2009 ).

One the other hand, some authors found relationships between swimming velocity, stroke length and stroke rate in freestyle and anthropometric characteristics of swimmers of both sexes. Pelayo et al. (2006) concluded that the differences in swimming velocity between male and female swimmers resulted from differences in stroke length, and that anthropometric characteristics affected swimming efficiency in women more than in men.

Hay et al. (1999) studied the effect of body roll on the hand path during the pull phase in the front crawl. In the authors’ model the swimmer’s hand was made to move in a plane through the shoulder, parallel to the sagittal plane of the rotating trunk. It was observed that when the body roll exceeds the amount necessary to produce the desired medial deviation of the hand, the swimmer must move the arm away from, rather than toward, the trunk’s midline.

Jürimäe et al. (2007) examined the influence of the energy cost of swimming, body composition and individual technical parameters on front crawl performance of pubertal and prepubertal swimmers. They found that the swimming technique, arm span and VO2peak appear to be major determinants of front-crawl swimming performance in young swimmers.

Polli et al. (2009) carried out an interesting analysis of average swimming speed (Av), stroke rate (SR) and stroke length (SL) in 50, 100 and 200m back crawl swimming tests. They concluded that the attainment of higher swimming speeds, regardless of swimming distance, should require an improvement of swimming technique and the SR/SL ratio.

Mason and Portus (2005) in their essay on biomechanical support in sport described an extremely useful method for examination and improvement of the swimmer’s symmetry, developed by the Australian Sport Institute. The method combines video footage from an underwater camera with diagrams of the swimmer’s hand force and body roll.

The swimming technique of a child differs from the technique of a mature swimmer, although the latter is often a teenager. This is common knowledge among swimming coaches. A developed technique in advanced swimmers has been described extensively in scientific literature and methodological manuals for swimming coaches. There are very few works, however, devoted to changes of swimming technique in the youngest swimmers. The present study attempts to assess changes in swimming technique in children swimmers.

The specific aim of the present study was to determine changes of selected angular characteristics and stroke cycle duration in front-crawl and back-crawl swimming in young novice swimmers. The study also attempted to assess the extent to which the selected swimming technique parameters can serve as markers of swimming performance efficiency.

## Material and Methods

### Participants

The participants included 11 children (9 boys, 2 girls) aged 8–13 years at the time of the first test (11.1 ± 1.64 years), who were taking part in swimming classes for beginners. The sample undertook seven swimming tests at three-month intervals. The seventh test took place 18 months after the start of the experiment. All participants were right-handed. The selection of children at such different ages was made in order to compare the development of swimming technique between younger and older children. Before the analysis of results the children were divided into two age groups: younger children aged 8–11 years, and older children aged 12–13 years. The differences between the two age groups are discussed in the final part of the results section.

The school swimming learning program comprised learning four styles taught in the following order: the back crawl, the front crawl, the breaststroke and the butterfly. The generally accepted methodology of swimming technique was applied. Swimming paddles were not used as learning aids in order to allow children to learn to “feel the water”, i.e. to learn to perceive the physical properties of water and use them effectively to improve swimming speed. Each technique was assessed during an intraschool swimming competition. Within the first four months of the training program the children learnt the basics of the four swimming strokes including starts, turns and general rules. Then they perfected their swimming skills by carrying out coordination exercises combining elements of various swimming techniques. Such complex movement sequences allowed faster and more precise mastery of particular swimming techniques.

Initially, the skill level of the examined children varied, thus the sample was divided into groups of more and less skilled subjects. Both groups were taught by the same swimming instructor. When after 10 months the level of swimming skills became similar in both groups and the groups were merged, elements of swimming training aimed at perfecting swimming technique and stroke length were introduced.

Three 45-min classes a week were conducted in a 25 meter swimming pool every week. Each class was preceded by a warm-up land drill. Initially, the children swam 500–600 m during one class, and the load increased to 1200–1400 m per class with the improved skill level, after 10-month training.

Moreover, the children attended non-swimming PE classes three times a week and once a week they took part in 30-minute dryland workouts for swimmers, before their swimming training in the water. The dryland exercises were general exercises and exercises with exercise bands aimed at improvement of elements of all swimming techniques. Overall their training was focused on the improvement of swimming technique. At this stage, the coaching aim was not to attain the best sport result, i.e. the fastest swimming time.

### Procedures

Each child was to swim 25 m backstroke, followed by 25 m front crawl. The movement technique was recorded by three cameras: 1. a fixed camera placed 25 cm underwater opposite the swimmer; 2. a fixed camera placed 25 cm above the waterline to the side of the swimmer; 3. a handheld camera covering the entire length of the pool. The recording with the use of cameras was extremely accurate at 50 frames per second. At this recording speed all movement details can be precisely analyzed, even at a very fast rate of locomotion.

### Measures

One whole properly executed swimmer’s movement cycle recorded by the cameras was then selected for analysis which involved geometric projections of angles of a child’s joints as seen along the line between the camera and the child. These projections will be referred to as “angles”. The projections of joint angles are seen by the instructor of swimming technique. Also the swimming coach, by analyzing swimming technique, considers the projections of these angles from his or her position on the side of the pool. This method of swimming technique analysis was used by Haljand (1997) , one of the most renowned researchers of the subject and by experts in swimming technique biomechanics ( Barbosa et al., 2011 ). Although these authors used other, more sophisticated assessment tools and carried out measurements of other angles, their assumptions point to a research direction that is worth following.

With the aid of the AVIIMAGE software the following movement technique parameters were measured:

1. The angle between the waterline and the line connecting the swimmer’s shoulder and hip axes (hereafter referred to as the angle of incidence) measured at two moments of the stroke cycle: during the arm’s entry into the water ( Pictures 1A and 1B ), and when the arm formed a 90 degree angle with the trunk axis during the recovery ( Pictures 1C and 1D ).
2. The smallest left and right elbow angle underwater in the front crawl and the back crawl ( Picture 2 ).
3. The maximal angle between the water surface and the line connecting the seventh cervical vertebra (C7) with the centre of shoulder joint (at the deepest point underwater) referred to as the shoulders roll ( Picture 3 ).
4. Stroke cycle duration divided into the underwater (power) phase and the recovery. The movement was recorded by the cameras at 25 frames a second, i.e. 1 frame lasted 0.04 s. The number of frames in a selected phase of movement multiplied by 0.04 was the time of this movement phase in seconds. The results were presented to the nearest 0.1 s.
5. In the period between the 4th and 7th tests stroke length was calculated for each participant as the mean length of the swimming stroke. The distance covered by the swimmer immediately after pushing off the pool wall was not taken into consideration.

### Analysis

The AVIIMAGE software package used to analyze the recorded images was developed by optoelectronics and IT specialists from the Poznań University of Technology. It permits a comprehensive analysis of angular and linear values from the recorded images.

For statistical analysis arithmetic means, medians, minimum and maximum, standard deviation values, the Wilcoxon signed-ranked test and U Mann Whitney test was used.

## Results

### Angle of incidence

The angle of incidence should amount to 8°–12° in the back crawl and 3°–10° in the front crawl ( Payton et al., 1997 , 1999 ; Jürimäe et al., 2007 ). Its size depends on the swimmer’s morpho-functional traits as well as on the phase of the stroke cycle. In the present study the angle of incidence was measured at two moments of the stroke cycle: at the swimmer’s arm’s entry in the water and when the arm formed a 90° angle with the trunk axis.

**Figure 1:**
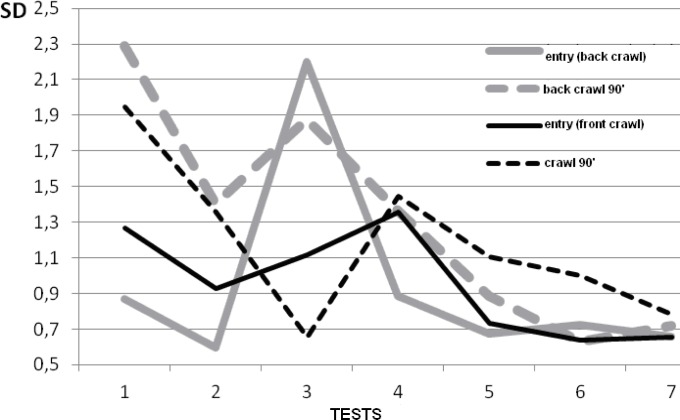
*
Standard deviation values for the angle of incidence
*

In both swimming styles the angle size was similar. It was also similar at the moment of the arm’s entry in the water in consecutive tests. The 90° angle decreased as the children improved their swimming technique ( Table 1 ). The difference, estimated with the Wilcoxon signed-ranked test, between the first and seventh tests in both styles was statistically significant (Z = 2.93, p < 0.005). It can be concluded that the children learned the correct, lower positioning of the head and the proper use of the arm’s propelling force to ensure the body’s movement forward rather than upwards. No relationship was found between the size of the angle of incidence and the participants’ age. The standard deviation values in the back crawl ranged from 0.5 to 2.3, and in the front crawl from 0.6 to 1.9 ( Figure 1 ). An interesting change in SD was observed as the children were acquiring more swimming skills. During the first three tests SD was high and diverse. Starting from the 4 ^th^ test the SD value decreased and became similar in the two swimming styles and two stroke cycle phases. During the 7 ^th^ test all four SD values were almost the same. This is evident of the swimming technique uniformization among all the examined children, resulting from the learning process. The question remains whether the matching of movement technique to the model is a positive phenomenon. It could as well be regarded as a manifestation of decline of individual characteristics of the swimming technique. After all, elite swimmers do display a high degree of technique individualization. Persistent adjustment of swimming technique to match the learning model and subsequent lack of individualization may not be entirely advantageous in view of children’s psychophysical traits.

### Elbow angle in the water

Bending the elbow in the propelling movement of the arm is an important component of both examined swimming styles. It prolongs the hand path and allows better positioning of the propelling hand surface against drag. The size of the elbow angle in the front crawl should be 90–120° in the front crawl, and 90–110° in the back crawl ( Payton et al., 1997 , 1999 ; Jürimäe et al., 2007 ).

In the examined children the elbow angle decreased, i.e. became less obtuse, systematically in all the tests for both swimming styles ( Table 2 ), which indicates the development of the children’s technical skills. The participants learned this important technical element successfully. The differences between the sizes of the angle in both styles were statistically non-significant.

The standard deviation values for the elbow angle ranged from 9.9 to 23.9, and varied for the right elbow and the left elbow. The clustering of results for the left arm was almost invariable in all seven tests. The standard deviation values for the right arm, however, decreased significantly in the 4 ^th^ test. This means that the attainment of the proper size of the elbow angle for the dominant arm can be learnt. The dominant arm can “feel the water” better, i.e. can adjust to drag more effectively. In consequence, better propulsion of the dominant arm can be achieved in the learning process.

No statistically significant differences between the elbow angles of both arms were noted in the back crawl tests. Starting from the 4 ^th^ test in the front crawl the differences between the values of the elbow angle between the left arm and the right arm were statistically significant. The results of the U Mann Whitney test ranged between 2.0 and 2.6, at p-value between 0.01 and 0.04.

### Maximal shoulders roll

The shoulders roll along the long axis of the body results from alternate propelling movements of the arms during swimming. In novice front crawlers an extra elevation of the shoulder can be also noted, which is caused by an excessive raising of the head during inhalation. In the front crawl, the optimal shoulders roll angle should fall between 40° and 50°, whereas in the back crawl it should amount to 40 – 45° ( Payton et al., 1997 , 1999 ; Jürimäe et al., 2007 ).

In the front crawl, in the first four tests, five children took breaths by turning the head to the left side, however, in the 5 ^th^ and 6 ^th^ tests only three children did it. In the 7 ^th^ test all the children took breaths on the right side. No statistically significant relations were found between the breathing side and the magnitude of shoulders roll in any tests.

**Figure 2:**
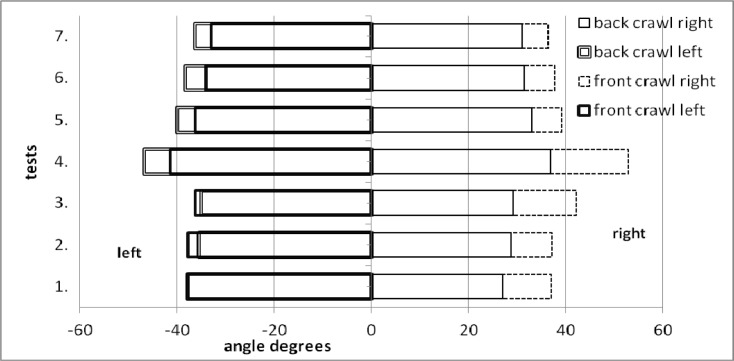
*
Arithmetic means of the maximal shoulders roll (7 tests, degrees)
*

In the back crawl all the left/right side differences were statistically significant (Z = 2.0–3.1, p = 0.00–0.04). The arithmetic means ranged between 35.1 and 46.8 degrees for the left side, and between 27.0 and 36.8 degrees for the right side. However, the shoulders roll mean values in the front crawl were 33.0–52.7 degrees. The trunk rotation was rather symmetrical in the front crawl as the left/right side differences measured with the U Mann Whitney test were statistically non-significant. The difference between the styles (back crawl/front crawl) was statistically non-significant for the left side and always statistically significant for the right side (Z = 2.3–3.1, p = 0.00–0.02). The shoulders roll to the right was bigger in the front crawl ( Figure 2 ).

The variability of standard deviation values was also characteristic for the right side in the front crawl. It can be assumed that it was related to the variability in the swimmers’ inhaling technique.

### Stroke cycle duration

Swimming strokes are cyclical, i.e. they consist of multiple, identical, repetitive movements. A stroke cycle is a movement that returns to its beginning and repeats itself in the same sequence.

In the analysis the stroke cycle was divided into two phases: power (underwater) phase and the recovery (above the water). In the back crawl the recovery was longer than in the front crawl. The duration of the power phase was similar in both swimming styles. In the back crawl both the power phase and the recovery became longer with learning the stroke ( Table 3 ).

The duration of the power phase ranged from 1.1 to 1.3 seconds in the back crawl and from 1.2 to 1.3 seconds in the front crawl. The recovery lasted from 0.5 to 0.8 seconds. The standard deviation was 0.27 for the back crawl and 0.43 for the front crawl. In the back crawl the time of both phases of the stroke cycle increased in subsequent tests; however, the differences were statistically non-significant.

In the front crawl the duration of both phases of the stroke cycle did not change. The differences in the length of the recovery in both styles (longer in the back crawl) in the 3 ^rd^ and 4 ^th^ tests were found to be statistically significant (U Mann Whitney test, p ≤ 0,01). It can be regarded as an indicator of a more efficient swimming technique.

### Stroke length

Stroke length is an index of swimming technique efficiency. It may reach its optimal value (neither too high nor too low) with the development of a relatively high swimming speed.

**Figure 3:**
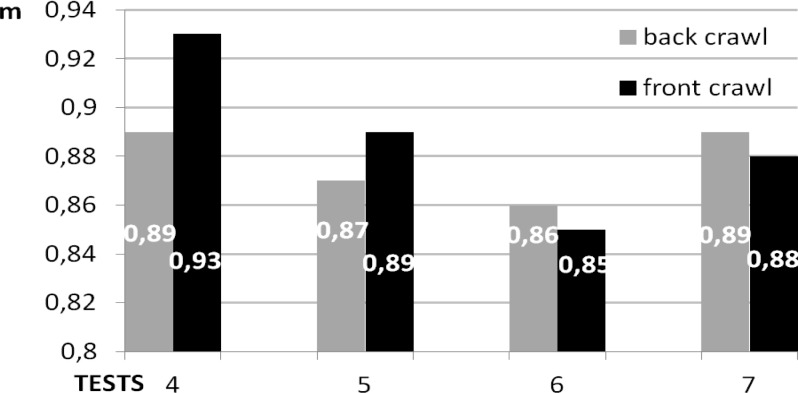
*
Stroke length (meters)
*

The numerical stroke length value was not really a significant factor of swimming performance in the present analysis ( Figure 3 ). It was slightly shorter in the 5 ^th^ , 6 ^th^ and 7 ^th^ tests than in the 4 ^th^ test. Neither were there any significant differences between stroke length results in the two studied swimming styles. It was not therefore an index of swimming efficiency.

In further analysis the children were divided into two age groups: younger children aged 8–11 years and older children aged 12–13 years. In the front crawl, the younger children displayed a greater angle of incidence, a more obtuse elbow angle and a bigger shoulders roll. The power phase in the first three tests was longer in older children, and starting from the fourth test it became similar to the power phase of the older children.

In the back crawl, the younger children also had a greater angle of incidence and a more obtuse elbow angle during the power phase. They also featured a longer recovery.

When the two youngest children, aged 8 years, were compared with the children from the age group of 12–13 years, the observed differences were more distinct in terms of angle of incidence and elbow angle values, especially in the front crawl. They also displayed a shorter stroke length. During the early tests the shoulders roll in younger children was bigger and the power phase longer. This is understandable since a longer movement of the arm underwater must cause a greater trunk rotation. These two interrelated parameters come close to the values obtained by the older children in the 4 ^th^ and 5 ^th^ tests. This progression illustrates the development of swimming techniques. In the back crawl, the 8-year-olds had also less bent elbows, smaller shoulders roll and shorter stroke length.

## Discussion

The available studies determine climber’s Swimming technique of adult swimmers has been discussed by numerous researchers, coaches and practitioners. It has been thoroughly described and analyzed with the use of high-tech underwater video recorders and software. Equally important is also the knowledge of the development of swimming technique in youth swimmers, which has a great impact on later individual technique characteristics in adult swimmers. In fact, the way swimming technique is learnt at a young age exerts a significant impact on the development of individual technical characteristics in adult swimmers in the future.

Swimming technique comprises numerous spatial and temporal movement characteristics. A swimming coach should therefore be equipped with precise diagnostic tools to be able to assess the progress of children and plan further development of swimming technique. The analysis of swimming technique parameters presented in this study can serve the purpose of such a diagnostic tool. The examined technique elements are easy to register, visually or electronically, and the gathered data can be easily processed. Such data analysis provides comprehensive information about the course of swimming movements in space and time as well as about the efficiency of swimming performance.

The obtained results provide swimming instructors with information about which characteristics change or not at any given stage of learning. The angle of incidence is an individual trait which being initially fairly diversified within a group of novice swimmers, should become very similar among youth within a short time. The elbow angle is a more individual characteristic and may remain on a different level among youth swimmers. The shoulders roll is a rather constant characteristic, which can be explained by its association with the range of motion in the shoulder joints and the level of skills exercised while inhaling.

The duration of phases of the stroke cycle is also a characteristic of low variability at any given swimming learning stage. However, the slightly longer power phase in younger children in the front crawl is natural and most likely results from their lower muscle strength and slower overcoming of drag. With the observed more obtuse elbow angle, this is understandable. The results of the study might suggest, however, that this difference would disappear after a few months of swimming technique development.

The longer recovery in the back crawl, observed in younger children is a negative phenomenon which increases the submergence of the body and drag. It is related to the lack of motor skills and does not improve during the observed period of swimming learning.

Swimming instructors should focus on the improvement of this parameter in order to develop earlier a more economical swimming technique.

The length of the stroke is related to the duration of a stroke cycle and the way of stroke performance. The results clearly confirm this observation and point to a low variability of this parameter within the sample.

Stroke length, that is the distance covered within one stroke cycle, is longer in swimmers with a well-developed and highly economical swimming technique.

In children, stroke length is, by necessity, shorter for two reasons: lower length parameters of the body, and less economical and still underdeveloped swimming technique. The youngest children under study demonstrated, in fact, the shortest stroke length.

Some of the obtained results pointed to certain regularities in the examined group of youth swimmers, others indicated individual differentiation. The results were not correlated with the children’s age, but with their individual physical traits and the duration of the learning process.

The analyzed swimming styles were the back crawl and the front crawl. Both strokes feature more or less steady propulsion and invariable angle between the swimmer’s body’s long axis and the waterline. A different analysis should be applied in case of the breaststroke and the butterfly which feature variable propulsion and the angle of incidence during each stroke cycle.

The results of the 3 ^rd^ and 4 ^th^ tests were markedly different from the results of earlier and later tests. The 3 ^rd^ and, in particular, the 4 ^th^ test can be thus regarded as turning points in the development of swimming technique by novice swimmers. These differences in the level of movement technique, in particular, maximal shoulders roll and angle of incidence, most likely result from changes in the training process since they appeared after the merger of the two groups of training children and increasing the training swimming distance twofold. The results of this change can serve as a warning to swimming coaches against increasing training loads too rapidly.

The study results also suggest when changes in children’s swimming technique can be expected. A positive swimming learning effect, i.e. decreasing of the angle of incidence, occurs already in the first months of swimming technique development. The subjects improve their movements quickly and achieve a similar positioning of the body as indicated by the decreasing SD values. Therefore, swimming coaches should expect the effects of correct trunk positioning against the waterline early.

Another parameter which is subject to positive changes during a short learning period is the elbow angle. Its adequate value during propulsion is necessary in learning the correct swimming technique. The study results suggest that this variable of swimming technique can be developed within a period of few months of swimming training.

The shoulders roll, duration of the stroke cycle and its phases and stroke length remain invariable in the studied period of children’s swimming learning. The results show that changes of these parameters come much later since they are related not only to the development of children’s motor skills but also come with the growth of the body, especially the length of the extremities.

The attained research results indicate a stepwise tendency in swimming technique learning, which is a well-known tendency among swimming coaches. The analysis of standard deviations is a significant marker of individualization or uniformity of swimming technique among the studied children.

## Conclusions

The results of the present study indicate the variability and phasing of learning of swimming technique by children. The results permit distinction of those elements of swimming technique of which development depends largely on technique learning (e.g. stroke cycle duration, elbow angle) from those that can be influenced by increasing training loads (maximal shoulders roll, angle of incidence). The study results also point to swimming technique elements which might be determined by one’s biological development as they require a greater body length (stroke length depends on the length of the extremities).

## Figures and Tables

**Picture 1:**
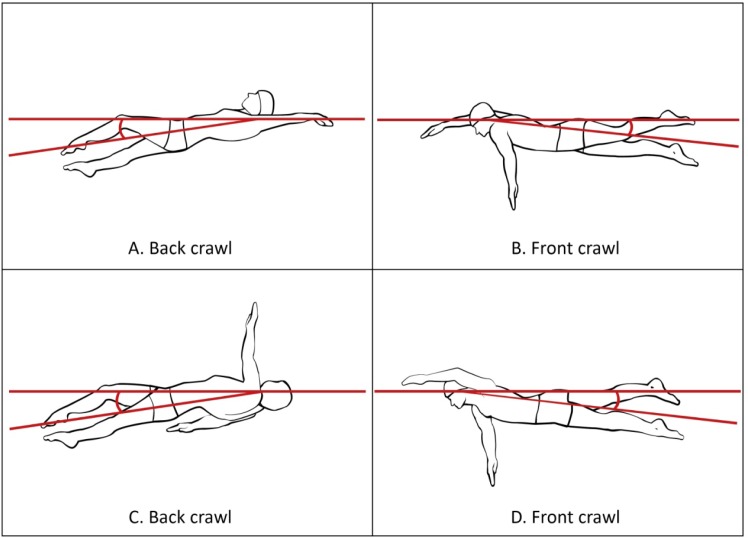
*
The angle of incidence at the arm’s entry in the water (A and B) and with the arm forming a 90° angle with the trunk axis (C and D)
*

**Picture 2:**
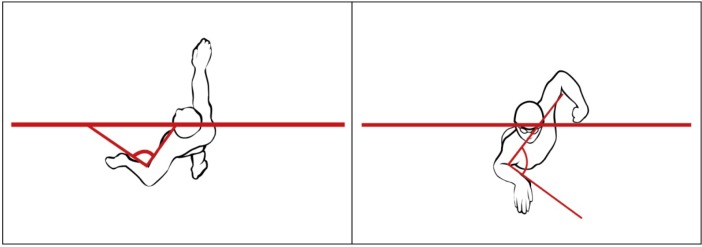
*
The smallest elbow angle in the back crawl and the front crawl
*

**Picture 3:**
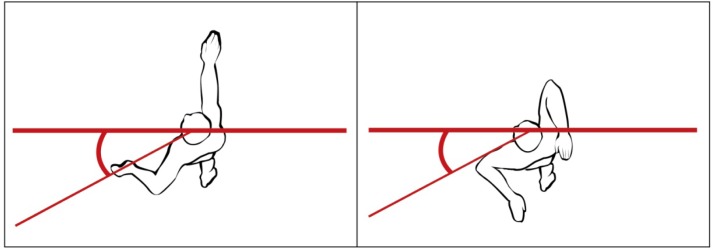
*
The shoulders roll in the back crawl and the front crawl
*

**Table 1:** *
The statistical characteristics of the angle of incidence values (angle degrees)
*

test number		1	2	3	4	5	6	7
back crawl								

arm’s entry in the water 90o angle	M	4.7	3.9	3.1	4.6	4.2	4.1	3.7
	min-max	3.3–6.2	2.6–4.76.1	1.9–9.84.7	2.7–5.75.7	3.2–5.35.8	3.2–5.35.4	2.7–5.05.0
	M	7.6	3.0–8.0	2.9–9.2	3.6–8.4	4.2–7.3	4.7–6.8	3.5–6.0
	min-max	5.6–14.3						

front crawl								

arm’s entry in the water 90o angle	M	4.7	4.3	3.0	4.2	4.2	4.4	4.1
	min-max	2.2–6.2	3.1–5.8	1.9–5.5	3.2–7.7	3.0–5.5	3.1–5.3	3.2–5.4
	M	6.8	6.1	5.2	6.3	5.4	5.2	4.8
	min-max	5.4–11.6	5.2–8.8	4.3–6.7	3.4–8.2	4.1–7.5	3.5–7.1	3.6–6.4

**Table 2:** *
The statistical characteristics of the elbow angle values in seven tests (angle degrees)
*

	test number	1	2	3	4	5	6	7

		back crawl						
left right	M	154	152	150	151	150	146	145
	min-max	109–188	119–167	117–170	116–168	116–174	115–169	111–165
	M	139	136	135	136	134	134	129
	min-max	124–179	112–179	95–172	103–174	106–169	105–168	107–161
	
		front crawl						
left right	M	149	149	149	148	146	145	144
	min-max	132–184	134–177	132–187	128–180	120–179	123–179	120–177
	M	156	146	135	140	133	133	130
	min-max	125–177	122–176	107–170	108–146	107–143	111–142	110–142

**Table 3:** *
Arithmetic means for the duration of the power and recovery phase (s)
*

test	1	2	3	4	5	6	7
back crawl							
power phase	1.1	1.2	1.2	1.3	1.3	1.3	1.3
recovery	0.6	0.7	0.7	0.8	0.8	0.8	0.8
front crawl							
power phase	1.3	1.2	1.3	1.3	1.3	1.3	1.3
recovery	0.6	0.6	0.5	0.6	0.6	0.6	0.6
